# The softening of Chinese digital propaganda: Evidence from the *People’s Daily* Weibo account during the pandemic

**DOI:** 10.3389/fpsyg.2023.1049671

**Published:** 2023-02-28

**Authors:** Chang Zhang, Dechun Zhang, Hsuan Lei Shao

**Affiliations:** ^1^School of Government and Public Affairs, Communication University of China, Beijing, China; ^2^Leiden Institute for Area Studies, Faculty of Humanities, Leiden University, Leiden, Netherlands; ^3^Department of East Asian Studies, National Taiwan Normal University, Taipei City, Taiwan

**Keywords:** China, propoganda, COVID-19, Weibo, state-run media

## Abstract

**Introduction:**

Social media infuses modern relationships with vitality and brings a series of information dissemination with subjective consciousness. Studies have indicated that official Chinese media channels are transforming their communication style from didactic hard persuasion to softened emotional management in the digital era. However, previous studies have rarely provided valid empirical evidence for the communicational transformation. The study fills the gap by providing a longitudinal time-series analysis to reveal the pattern of communication of Chinese digital Chinese official media from 2019 to 2022.

**Method:**

The study crawler collected 43,259 posts from the *People’s Daily’s* Weibo account from 2019 to 2021. The study analyzed the textual data with using trained artificial intelligence models.

**Results:**

This study explored the practices of the *People’s Daily’s* Weibo account from 2019 to 2021, COVID-19 is hardly normalized as it is still used as the justification for extraordinary measures in China. This study confirmed that *People’s Daily’s* Weibo account posts are undergoing softenization transformation, with the use of soft news, positive energy promotion, and the embedding of sentiment. Although the outburst of COVID-19 temporarily increased the media’s use of hard news, it only occur at the initial stage of the pandemic. Emotional posts occupy a nonnegligible amount of the *People’s Daily* Weibo content. However, the majority of posts are emotionally neutral and contribute to shaping the authoritative image of the party press.

**Discussion:**

Overall, the *People’s Daily* has softened their communication style on digital platforms and used emotional mobilization, distraction, and timely information provision to balance the political logic of building an authoritative media agency and the media logic of constructing audience relevance.

## Introduction

Digital media play a vital role in enabling the government to disseminate information, raise public awareness, and influence the public’s perception of events (Durant and Lambrou, 2009). Therefore, governments are increasingly prioritizing social media and creating official accounts (Huang and Lu, 2017), which has blurred the boundaries among news, propaganda, and entertainment; China is no exception. The digitalization of official media sources has led to a change in communication styles (Huang and Lu, 2017; Shao and Wang, 2017). For instance, Chinese state-run media has adopted a more nuanced approach to forming social consensus and increasing social cohesion than simply applying discursive coercion (Zhang, 2011; Edney, 2014). This led Huang (2015) to suggest that the Chinese state-run social media strategy involves hard and soft propaganda. Hard propaganda is biased information that frames the ruling party positively without mobilizing emotions, whereas soft propaganda involves emotional mobilization (Huang, 2015; Xia, 2020; Mattingly and Yao, 2022). Multiple studies have indicated that official Chinese propaganda outlets have achieved a delicate balance between positive and negative emotions to evoke emotional resonance and creative engagement (Kong, 2016; Fang and Repnikova, 2018; Gao and Wu, 2019), especially during the COVID-19 pandemic (Zhang, 2022).

During the early stages of COVID-19, the Chinese government’s delayed reaction incurred criticism from around the world. Several studies have explored the Chinese government’s propaganda during COVID-19 (Zhang, 2022; Zhang and Jamali, 2022). Studies have suggested that the Chinese government has triggered a sense of nationalism on social media by mobilizing emotion (Zhang, 2022) and positively framed the Chinese government (Zhang and Jamali, 2022; Zhang and Xu, 2022). Although China’s digital propaganda is well documented, most studies have examined China’s digital practices through case studies or qualitative observations and interviews rather than longitudinal time-series analysis. Therefore, this study systematically examined the digital practices and propaganda of Chinese official media from 2019 to 2022. Specifically, this study explored how the Chinese official media used propaganda on Weibo during this period and how COVID-19 influenced their communication strategies. The study evaluated hard news vs. soft news, the news topics covered, and the emotional content of the news to analyze the transformation of Chinese digital propaganda and the effects of the pandemic.

This study examined the 2019, 2020, and 2021 Weibo posts of *People’s Daily* by using trained artificial intelligence models. The study determined that soft news dominated the *People’s Daily* Weibo posts, although the frequency of hard news exceeded that of soft news during the early stages of the COVID-19 pandemic. This study also determined that although emotional mobilization occurred on Weibo, most posts were nonemotional in an apparent attempt to maintain a sheen of objectivity. China’s official social media posts create interaction with the public, and outlets switched to releasing authoritative information (hard news) during the early stages of COVID-19 to reduce panic and promote the stability of public sentiment.

## Changing landscape of the Chinese digital media environment

Chinese media serve as the mouthpiece of the Chinese Communist Party (CCP; Duan and Takahashi, 2017), which are widely regarded as a party–state political apparatus (Duan and Takahashi, 2017; Qi, 2018; Zhang, 2021) owned, funded, and monitored by the Chinese government (Chan and Qiu, 2001). Jian and Liu (2018) suggested that Chinese media only distributed propaganda because they were fully subsidized by the government from 1949 until the 1980s. Zhang (2021) framed Chinese official media as affiliate organizations of the Chinese government designed to spread the government’s fundamental ideologies (Duan and Takahashi, 2017). However, since the development of the economy and the commercialization of the Chinese media in the 1990s, the media have enjoyed greater freedom (Tan, 2012). Although Chinese media have adopted marketization techniques such as selling advertising, they remain under government supervision (Xu, 2016). Therefore, Wright et al. (2020) suggested that Chinese state media struggle to balance being the government mouthpiece and being a news organization.

Internet and social media use have become widespread in China. According to Statista (2018), the number of Weibo users increased substantially, from 63 million in 2010 to 350 million in 2018. Studies have suggested that social media plays a key role in Chinese politics (Schneider, 2018; Zhang, 2021; Zhang and Xu, 2022). Therefore, the Chinese government has prioritized regulating social media and the internet (Xu and Albert, 2017). Social media accounts are subject to government monitoring, and account owners must self-censor before posting (King et al., 2017). In May 2010, the Information Office of the State Council of the People’s Republic of China published a white paper on implementing a more restrictive redline for all internet users, which was followed by the Public Pledge on Self-Regulation and Professional Ethics for the Chinese Internet Industry (Xu and Albert, 2017). The Chinese government usually punishes noncompliant social media users (Ruan et al., 2021). The control of social media has become a part of state stabilization, thereby demonstrating the will to power of the Chinese authorities. Any content designed to enflame or encourage collective movement and social mobilization is the first-line target of government censorship compared with negative posts and even acrimonious criticisms of the state and its leaders and policies (King et al., 2017). Therefore, the Chinese government leaves limited space for the public to participate in politics to appease injustice (King et al., 2017; Fang and Repnikova, 2018). Yan (2020) determined that the Chinese government uses hard power (censorship) and soft power (reshaping public opinion by promulgating ideologies such as nationalism) together to make the internet a digital extension of Chinese-governed geopolitical territory.

## China state media’s digitalization

According to the 47th China Statistical Report on Internet Development (CNNIC, 2021), approximately 740 million Chinese internet users access news online. Therefore, the prosperity of the internet and social media provide internet users with options other than state-owned media to receive information (Xin, 2018). Therefore, traditional state-owned media face challenges and must publish tailor-made content to increase their audience and influence while promulgating party viewpoints and policies (Li, 1998, p. 315). Chinese state media use social media platforms to confront these challenges (Huang and Lu, 2017). For instance, new organizations publish news on their websites and Weibo and WeChat accounts (Wu and Pan, 2021). Chinese state media have moved to social media and transitioned from didactic hard persuasion to softened emotion management.

Social media offers an opportunity to change the state media’s communication style (Zhu, 2017). Studies have noted that official social media accounts follow the social media communication logic (Han and Qi, 2019; Shi and Tong, 2020) of competing for attention (Durant and Lambrou, 2009). The traditional relationship between audiences and news organizations is changing because more people are accessing news through mobile devices. Sharing, commenting, and liking news have already altered the assumption of the passive audience in the traditional model of a one-way flow of information (Andersen et al., 2019, p. 2480). Social media is designed for public use, so content should attract the public (Huang and Lu, 2017). Therefore, to survive fierce competition, news organizations must understand the public’s needs to retain interest in the news (Nelson, 2019). To achieve this, Chinese state media on digital platforms have adopted simple methods of expression (Huang and Lu, 2017). Chinese official media have shifted from the mouthpiece model to a user-oriented model, with the language shifting from formal to informal to increase viewership (Huang and Lu, 2017; Shao and Wang, 2017). Therefore, the social media accounts of Chinese state media have changed the old model of “you broadcast, and I watch” to a model that encourages interaction with audiences (Huang and Lu, 2017, p. 3). However, although news media have adopted digitalization, this has failed to transform their official, exclusive, and professional nature (Wu and Pan, 2021; Zhang, 2021). Liu and Zhou (2011, p. 218) noted that although the Weibo accounts of Chinese state media serve as a tool to capture the public voice, a platform to collect sources, and a channel to obtain social news, they also serve as propaganda tools to facilitate the spread of positive news and manage crises. Therefore, the social accounts of Chinese state media have multiple identities: attention-hungry social media that must survive in a competitive (Nye, 2008) marketplace, the propaganda machine of the CCP, and a gatekeeping news organization (Thussu, 2018).

## Digital practice and digital propaganda in China

Traditionally, “propaganda” has referred to “an attempt to transmit social and political values” (Kenez, 1985, p. 4). In other words, propaganda refers a means of manipulation of information or significant symbols to influence public opinion (Lasswell, 1927). The Chinese government is well known for using the mass media to conduct propaganda (Thussu, 2018). Shambaugh (2007, pp. 26–27) noted that Chinese propaganda involves “mass mobilization campaigns; the construction of ideological and educational ‘models’ to be emulated; the control of the content of newspaper articles and editorials; development of a nationwide system of loudspeakers that reached into every neighborhood and village; the domination of the broadcast media.” Chinese propaganda is used to consolidate the government’s control over society, stabilize the state, and defend national interests through self-promotion and self-advocacy (Li, 2019, p. 145; Wang, 2012; Zhang and Jamali, 2022). Chinese official media produce a massive amount of content to increase visibility to the public (Xu and Wang, 2022). Studies have suggested that Chinese media propaganda uses social media logic to publish information that appeals to the public (Xia, 2020; Zeng and Li, 2021; Xu and Wang, 2022). For instance, Chinese state media on digital platforms often post soft news rather than hard news (Liang, 2019; Zeng and Li, 2021).

On social media, Chinese state media often report news about economics, international affairs, and environmental problems in a positive tone, whereas civil rights, politics, the military, and terrorism appear less often and have a negative tone (Liang, 2019). Liang (2019) indicated that Chinese official media on digital platforms uses soft power to increase the visibility and positivity of their policies. Scholars have also suggested that the state media following social media logic also create two kinds of propaganda: hard propaganda and soft propaganda (Mattingly and Yao, 2022). Hard propaganda refers to news reports that are inaccurate, exaggerated, fabricated, favorable to China, and antagonistic toward its opponents without emotional mobilization (Huang, 2015). In this sense, hard propaganda is designed to “signal the [ruling party’s] strength in social control and [its] capacity to meet potential challenges” (Huang, 2015, p. 435). Hard propaganda usually frames the ruling party positively, promotes mainstream narratives and ideologies, exaggerates leaders’ actions, and presents disinformation regarding antagonists (Xia, 2020; Mattingly and Yao, 2022). Since Xi Jinping came to power, several studies have explored the transformation of Chinese propaganda from heavy-handed hard persuasion to more refined, emotionally resonant, and esthetically appealing soft propaganda (Huang, 2015; Statista, 2018;Zou, 2021; Mattingly and Yao, 2022). According to Huang (2018), soft propaganda is distinguished by its subtle and sleek political persuasion style by contrast with hard propaganda’s crude and heavy-handed style. Although no settled definition of soft propaganda exists, soft propaganda generally refers to propagandistic content packaged in entertaining formats with emotional mobilization (Zou, 2021; Mattingly and Yao, 2022). Therefore, soft propaganda involves a shift from politicized indoctrination to entertainment-oriented media content production. Lu and Pan (2021) determined that the Chinese government’s digital propaganda tends to include nonpolitical entertaining news content to attract viewers. For example, the propaganda apparatus increasingly synthesizes elements from visual subculture to appeal to the younger generation well-versed in popular culture (Chang and Ren, 2018). Zou (2021) noted that digital propaganda in China not only demonstrates playfulness but also mobilizes emotions through interaction. Therefore, emotional turns constitute another pattern of China’s soft propaganda transformation. Chinese soft propaganda generally arouses fear, anger, and grievances by mobilizing the public’s sense of nationalism (Huang, 2015; Mattingly and Yao, 2022). Zhang and Jamali (2022) explored how the Chinese media on Weibo report on Western vaccines to mobilize negative emotions toward foreign countries. Research has also revealed the mobilization of positive emotions in Chinese propaganda (Yang and Tang, 2018). Wang (2020) analyzed emotional mobilization through the lens of positive energy, which captures the government’s endeavors to promote optimism, confidence, and patriotism while containing negative emotions (Wang, 2020). The promotion of positive energy, according to Yang and Tang (2018), is used to align the public’s emotions with the ideological and value systems of the CCP party-state. Hird (2018) contextualized the positive energy campaign within a neoliberal emotion management regime. In this regime, the pursuit of happiness is privatized, shirking the responsibility of the public sector or private institutions. Analysis of Hird (2018) considered affective biopolitics in Foucauldian terms, which relates to regulating feelings and attitudes in line with the objectives of emotional governance. Zou (2021) used interviews to determine that official media staff members embed emotions into news media production in the service of ideological governance through institutional routines, especially during national scandals (Wang, 2020).

China’s poor handling of the COVID-19 pandemic in the early stages received considerable domestic criticism. Zhang (2022) determined that mouthpiece *People’s Daily* promoted positive actions from ordinary people to turn negative emotions of grief into positive national solidarity and distract the public, thereby changing the narrative. A discourse analysis conducted by Wang (2020) revealed that the Chinese government presented itself as a savior of the Chinese people during domestic national crisis by punishing officials, informing the public of effective measures, and refuting rumors that had spread on social media. Chen and Wang (2019) suggested that these discourses discipline citizens into being docile subjects who internalize state interests and have the desired emotional responses to current events. Thus, Chinese state media on digital platforms changed their practices to increase their audience and mobilized emotions to conduct soft propaganda during the COVID-19 pandemic (Zou, 2021; Mattingly and Yao, 2022).

## Data collection

This study used Weibo posts from *People’s Daily* to explore the Chinese official media’s digital propaganda practice. *People’s Daily* is well known as the mouthpiece of the Central Committee of the CCP, which is the center of state power (Wu, 1994). Therefore, *People’s Daily* as a government organ determines the agenda for the rest of the media (Luther and Zhou, 2005; Song et al., 2016). Some scholars have noted that *People’s Daily* could represent or at least reflect the standpoints of the Chinese government or China’s top leadership (Wu, 1994; Mao, 2014). The official Weibo account of *People’s Daily* has more than 116 million followers, making it a highly influential and authoritative account (Dong et al., 2020). Therefore, the official Weibo account of *People’s Daily* is most representative of the digital practice and digital propaganda of the Chinese official media. This study used a web crawler to collect Weibo posts published by *People’s Daily* between January 1, 2019, and December 31, 2021. We used this 3-year period because it contains three stages: before the pandemic (2019), the middle of the pandemic (2020), and after the pandemic (2021). This enabled us to explore the digital practices of *People’s Daily* and the changes that occurred after the pandemic began. The crawler collected 43,259 posts for our sample.

## Research design

**Figure 1:**
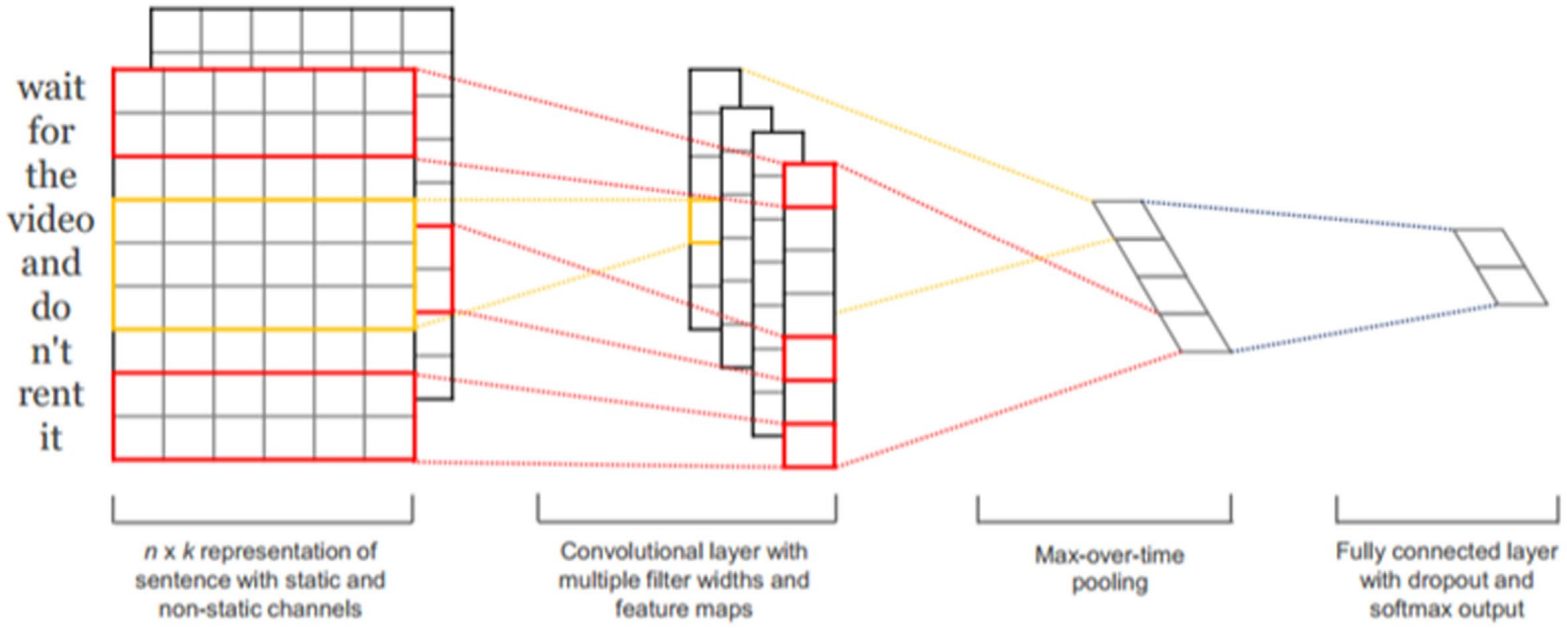
Convolutional neural network (CNN) structure (Kim, 2014).

**Figure 2:**
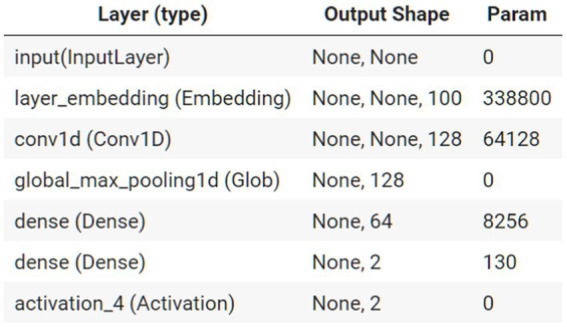
Text-CNN structure.

After collecting the posts, we randomly read 3,000 posts from each year and marked them as hard news or soft news (hard news = 1; soft news = −1) and as emotional or nonemotional (emotional = 1; nonemotional = −1).^1^ Hard news refers to major breaking news about top leaders, major issues, or disruptions in daily life (Patterson, 2000) that should be urgently reported, such as information related to COVID-19 (number of cases and epidemic prevention policy announcements), whereas soft news is “typically more sensational, more personality centered, less timebound, more practical, and more incident-based than other news,” covering human-interest stories about ordinary Chinese citizens (Patterson, 2000). We used the Python text–convolutional neural network (CNN) algorithm in the kashgari package (Eziz, 2019). CNNs have been commonly applied to analyze visual imagery. They can extract features of images and magnify these characteristics (Krizhevsky, 2012). They use a kernel to see-around-point and determine the partial features in the area. This study used a CNN to extract text features and classify them. For example, with a kernel size of 4, the text “新的一年静下心来读书吧” (“calm down and read a book in the new year”) would be broken down into “新的一年” (new year), “的一年静” (“calm new year”), “一年静下” (“calm down in the new year), and “全年静下心” (calm down all next year). Groups of four characters can be analyzed to connections between segments, which can be used to extract meaning from sequential data. Most combinations are not meaningful, but the algorithm can identify meaningful segments and increase their weight to build a model. Compared with a similar method, the bag of words, text-CNN is more appropriate for the Chinese language (Huang and Shao, 2020). Both of our main models achieved higher than 80% accuracy for the test set (Figures 1, 2; Tables 1, 2).

**Table 1:** Evaluation of model for hard and soft news.

Model_H/SNews	Precision	Recall	f1-score	Support
SoftNews	0.7640	0.8608	0.8095	158
HardNews	0.8571	0.7586	0.8049	174
Accuracy			0.8072	332
Macro avg	0.8106	0.8097	0.8072	332
Weighted avg	0.8128	0.8072	0.8071	332

**Table 2:** Evaluation of model for emotional and nonemotional news.

Model_emotion	Precision	Recall	f1-score	Support
No-emotion	0.8696	0.8475	0.8584	236
Emotion	0.6897	0.7273	0.7080	110
Accuracy			0.8092	346
Macro avg	0.7796	0.7874	0.7832	346
Weighted avg	0.8124	0.8092	0.8106	346

This study used latent Dirichelet allocation (LDA) to explore popular topics in *People’s Daily* Weibo posts. LDA is an unsupervised clustering tool that can be used to determine latent topics that we are not certain about in the first stage. We used the Python gensim package (Rehurek and Sojka, 2011) and identified 100 feature words for each topic to name the clusters. We selected four topics: social news (Chinese domestic social news, 11,370 posts), Chinese political news (China-related political news, 10,990 posts), world news (8,374 posts), and positive-energy posts (including motivational articles, 12,525 posts) (Table 3).

**Table 3:** The 20 most frequent words/phrases of each topic.

Naming by authors	Society	World news	Chinese news	Positive energy posts
Natural clustering	Topic1	Topic2	Topic3	Topic4
1	社会 (Society)	疫情 (Pandemic)	病例 (Case)	习近平 (Xi Jingping)
2	检测 (Test)	北京 (Beijing)	确诊 (Confirm)	生活 (Life)
3	学生 (Student)	美国 (United States)	新增 (New case)	世界 (World)
4	孩子 (Child)	香港 (Hong Kong)	报告 (Report)	加油 (Fighting)
5	核酸 (PCR test)	防控 (Prevent pandemic)	死亡 (Death)	我国 (Our country)
6	全球 (Worldwide)	发布 (Release)	累计 (Total)	明天 (Tommorow)
7	上海 (Shanghai)	情况 (Situation)	输入 (Import)	你好 (Hello)
8	男子 (Man)	新闻 (News)	患者 (Patient)	努力 (Work hard)
9	女子 (Woman)	发布会 (Press conference)	湖北(Hubei)	小时 (Hour)
10	民警 (Policeman)	人员 (People)	境外 (Overseas)	人生 (Life)
11	高考 (Gaokao)	回应 (Response)	本土 (Domestic)	疫苗 (Vaccine)
12	北京 (Beijing)	地区 (Region)	现场 (Site)	发展 (Develop)
13	首次 (First)	外交部 (Ministry of foreign affairs)	感染 (Affect)	周年 (Anniversary)
14	学校 (School)	组织 (Organization)	新冠 (Covid)	未来 (Future)
15	山东 (Shandong)	介绍 (Introduce)	无症状 (Asymptomatic)	希望 (Hope)
16	河南 (Henan)	关注 (Attention)	医院 (Hospital)	夜读 (Night reading)
17	抗疫 (Control pandemic)	调查 (Investigate)	感染者 (Infected)	学习 (Study)
18	医生 (Doctor)	发言人 (Spokesman)	祖国 (Homeland)	东京 (Tokyo)
19	浙江 (Zhejiang)	相关 (Related)	英雄 (Hero)	感恩 (Grateful)
20	老人 (Elder)	病毒 (Virus)	致敬 (Salute)	微博 (Weibo)

**Figure 3:**
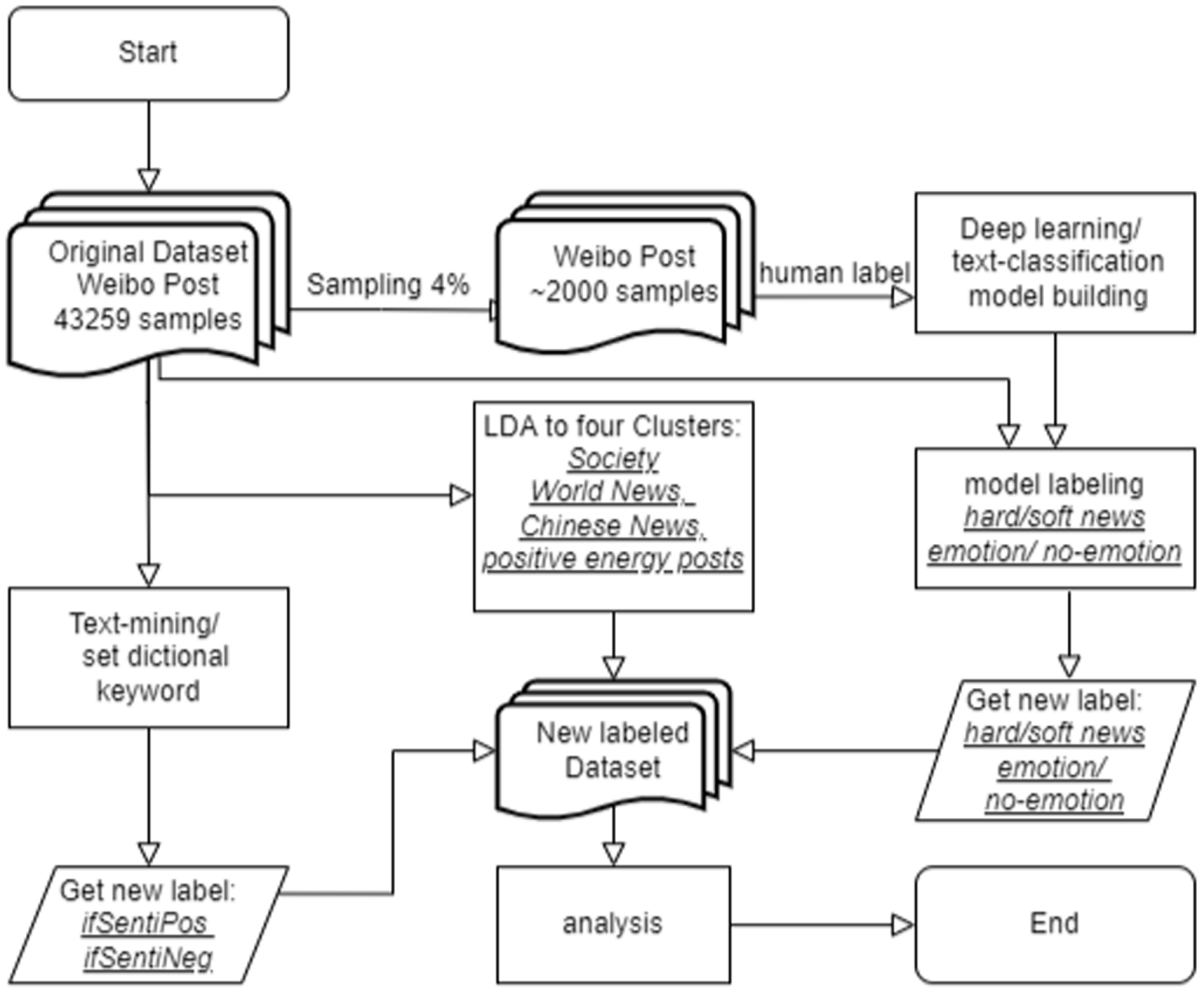
Research workflow.

This study used the traditional dictionary method to annotate posts with positive or negative words and perform sentiment analysis. Authors with two extra coders defined a dictionary of positive sensitivity and negative sensitivity (Supplementary Appendix A). Each post that had a word in these dictionaries was labeled as 1 and 0 otherwise. We obtained the labels “ifSentiPos” (0 for 32,276 posts and 1 for 10,983 posts) and “ifSentiNeg” (0 for 40,220 posts and 1 for 3,039 posts; Figure 3).

## Softening of Chinese digital propaganda I: Soft news replacing hard news

**Figure 4:**
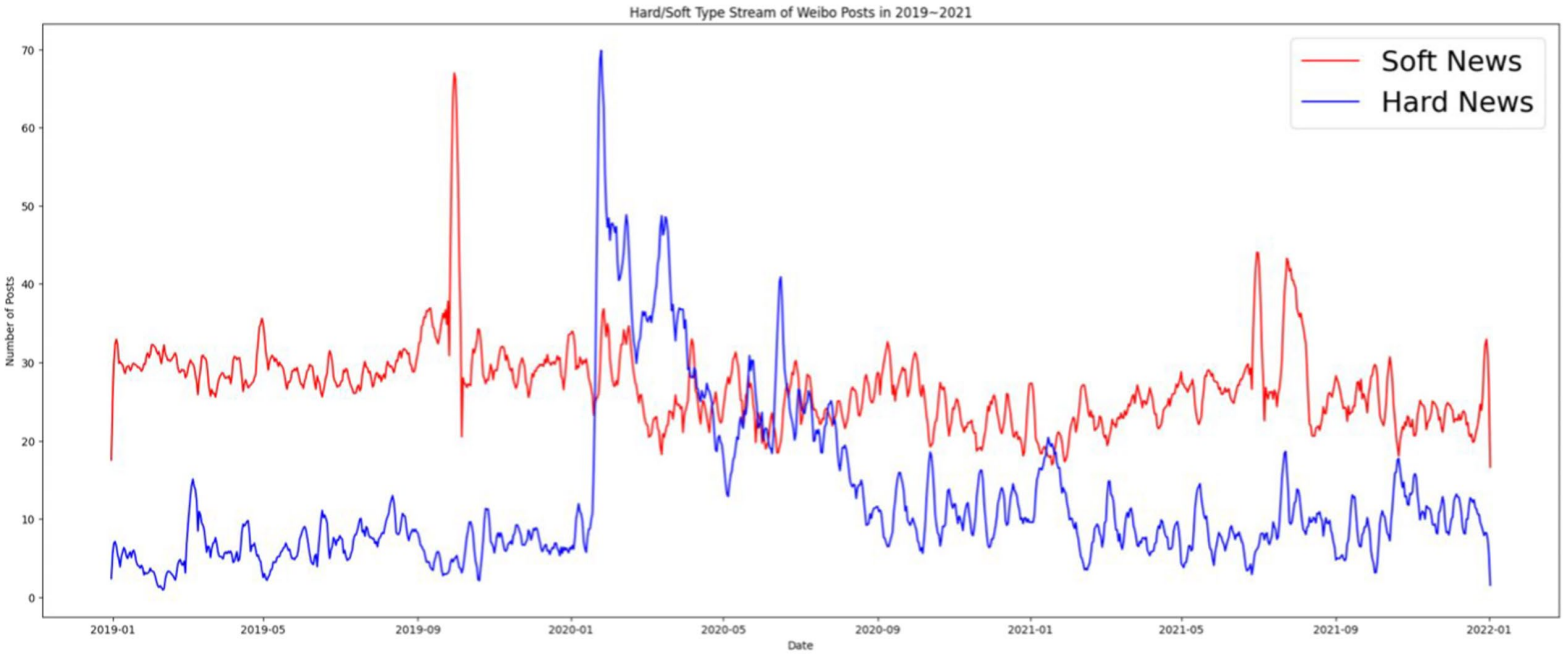
Hard and soft news on *People’s Daily* Weibo account from 2019 to 2021.

Since the 2000s, scholars have acknowledged the political relevance of soft news. Baum (2002) revealed that political information packaged as soft news and therefore involving entertainment, sensationalization, and a human interest lens tends to be more appealing to apolitical audiences. Although exposure to soft news may not increase an audience’s knowledge of political reality (Prior, 2003), it effectively reinforces the audience’s existing attitudes toward foreign policy for both isolationists and internationalists (Baum, 2003). This study determined that Chinese digital propaganda increasingly involves soft news. For the majority of 2019–2021, soft news was used as the main format of posts on the *People’s Daily* Weibo account. The only exceptions were between January 2020 and July 2020, when the number of hard news posts exceeded that of soft news posts twice. The first surge of hard news posts followed the outbreak of COVID-19 in China (starting in late January), and the number gradually decreased to a low in May, which marked the first stage of success in China’s battle with COVID-19 because all provinces, including the epicenter of Hubei Province, released the first-level public health emergency response on May 1, 2020. The number of hard news posts exceeded that of soft news posts in June but decreased in July. This period corresponds to a short outbreak of COVID-19 in Beijing, which started from the first detected case in the Xinfadi Wholesale Market on June 5, 2020, to July 10, 2020, when no new cases were detected (Wang et al., 2021). After the outbreak in Beijing was brought under control in July, soft news maintained its dominance over hard news until the end of the study period. The findings have two implications. First, soft news was generally the dominant format of the *People’s Daily* Weibo posts. Second, in the time of crisis, the official media assumed the role of the legacy media, conveying politically relevant information through rational and nonemotional forms. The *People’s Daily* Weibo account demonstrated its identity as the party press by conveying authoritative news to the public and pass public opinion up to the political authority, the CPC government (Figure 4).

## Softening of Chinese digital propaganda II: Dominance of positive-energy posts

**Figure 5:**
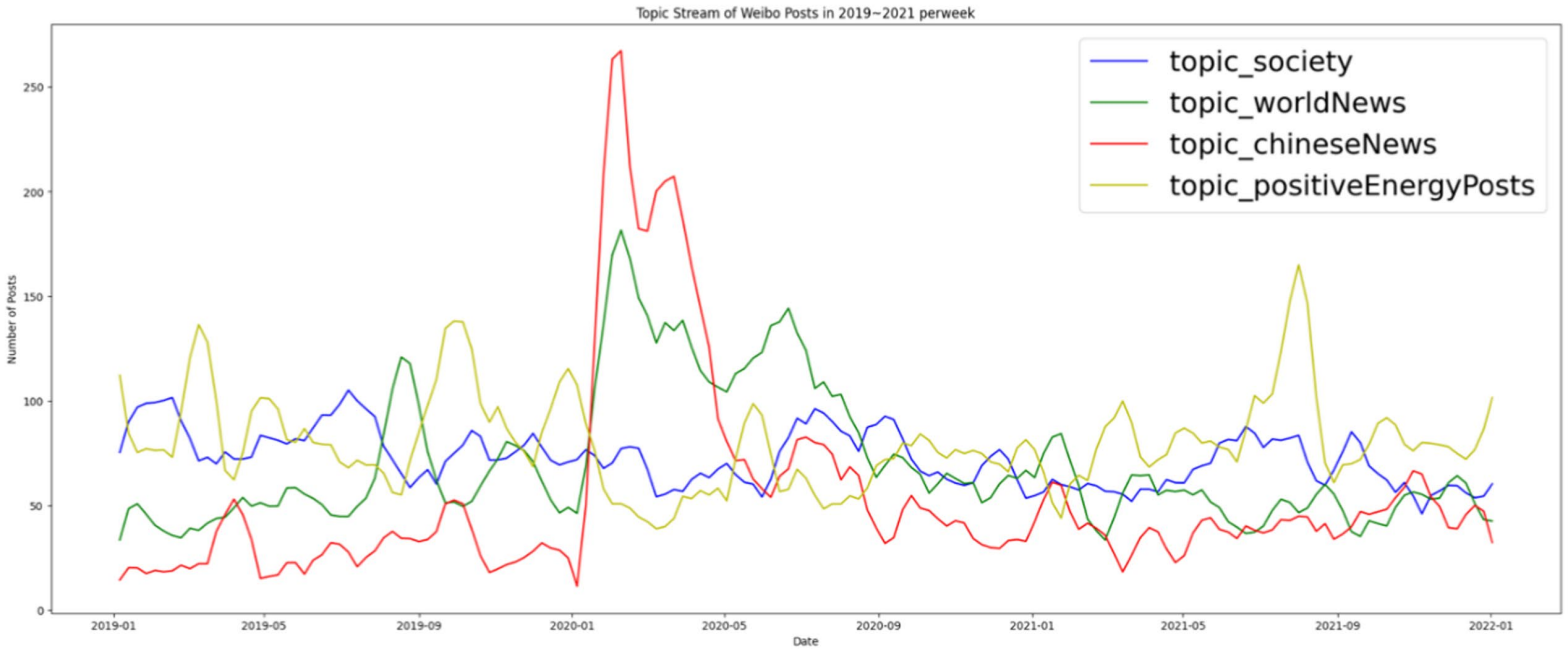
Topics covered on *People’s Daily’s* Weibo account from 2019 to 2021.

Emotional management was the main task of the *People’s Daily* digital account, and positive energy was the most prevalent topic in posts. The next most prevalent was social news about depoliticized social issues. Political news was the least prevalent, with world news posts outnumbering Chinese domestic political news posts. This suggests that the *People’s Daily’s* social media account prioritized interaction over information; that is, they prioritized dialogic interaction with the audience over informing the public of global and domestic political news. After the pandemic started, the number of news posts rose dramatically, especially those about Chinese politics and world news. This is consistent with the findings of Zhang (2011, p. 60), who indicated that the Chinese state media endeavored to increase its relevance by privileging market logic at least as much as they did party logic to spread the views of the government. The public health crisis temporarily changed *People’s Daily* preferences, leading to them prioritizing informing the public over audience engagement (Table 4; Figure 5).

**Table 4:** Correlation between hard and soft news and topics (*p* < 0.001; significant correlation with chi-square contingency on news type by post topics).

Topics\news_type	Soft	Hard	Sum	Soft_%	Hard_%
Society	9,907	1,463	11,370	87.13	12.87
World news	5,038	5,952	10,990	45.84	54.16
Chinese news	3,909	4,465	8,374	46.68	53.32
Positive energy posts	10,694	1831	12,525	85.38	14.62

**Figure 6:**
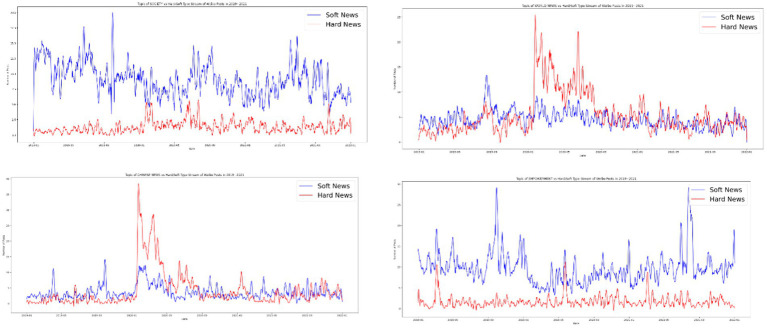
Hard news vs. soft news by topic over time; upper left, society; upper right, world news; lower left, Chinese political; lower right, positive energy.

After establishing the dominance of soft news and positive posts during normal times and the surge of hard news and informational posts at the peak of the pandemic, we investigated how the *People’s Daily* matched topics with formats. A cross-examination revealed that the majority of soft news focused on social news (87.13%) or promoting positive energy (85.38%), although a nonnegligible amount of world news (45.84%) and Chinese news (46.68%) was covered in the form of soft news. More political news, including those relating to both international affairs and domestic politics, was packaged as hard news. Soft news was the main format for social news from *People’s Daily* and posts to spread positive energy, and both soft and hard news were used when domestic political news and foreign news were the subjects. During the initial stages of the pandemic, when hard news superseded soft news in the *People’s Daily’s* Weibo posts, more social news was expressed in the form of hard news. The same pattern was observed for Chinese domestic political and foreign news. Although positive-energy posts tended to be soft news, the outbreak of COVID-19 led to several positive-energy posts in the form of hard news (Figure 6).

This study determined that the pandemic prompted the *People’s Daily* Weibo account to pivot to being a traditional media outlet that disseminates hard news. However, COVID-19 did not have a long-term effect on communication strategies, and soft news regained dominance shortly after the pandemic.

## Softening of Chinese digital propaganda III: Emotionalization of the party press’s digital accounts

**Figure 7:**
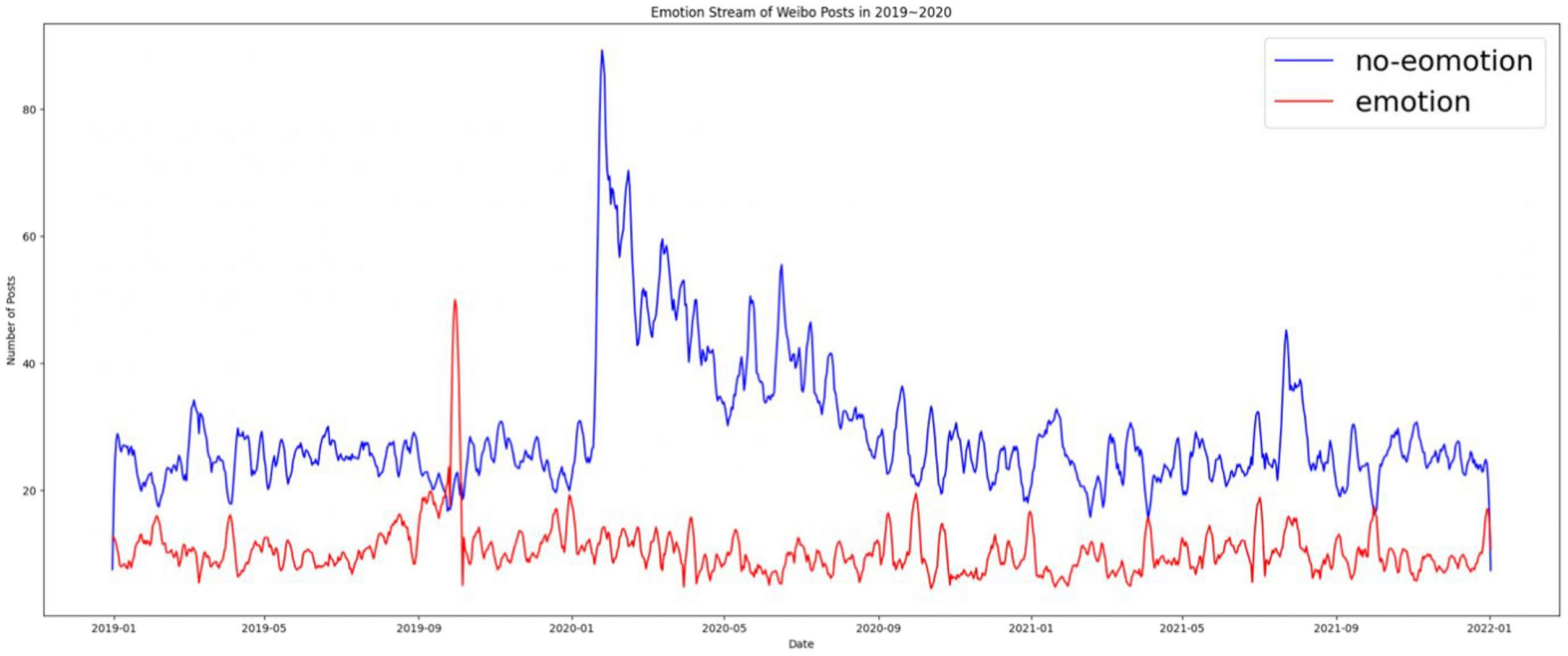
Emotional and nonemotional news on *People’s Daily* Weibo account from 2019 to 2021.

**Figure 8:**
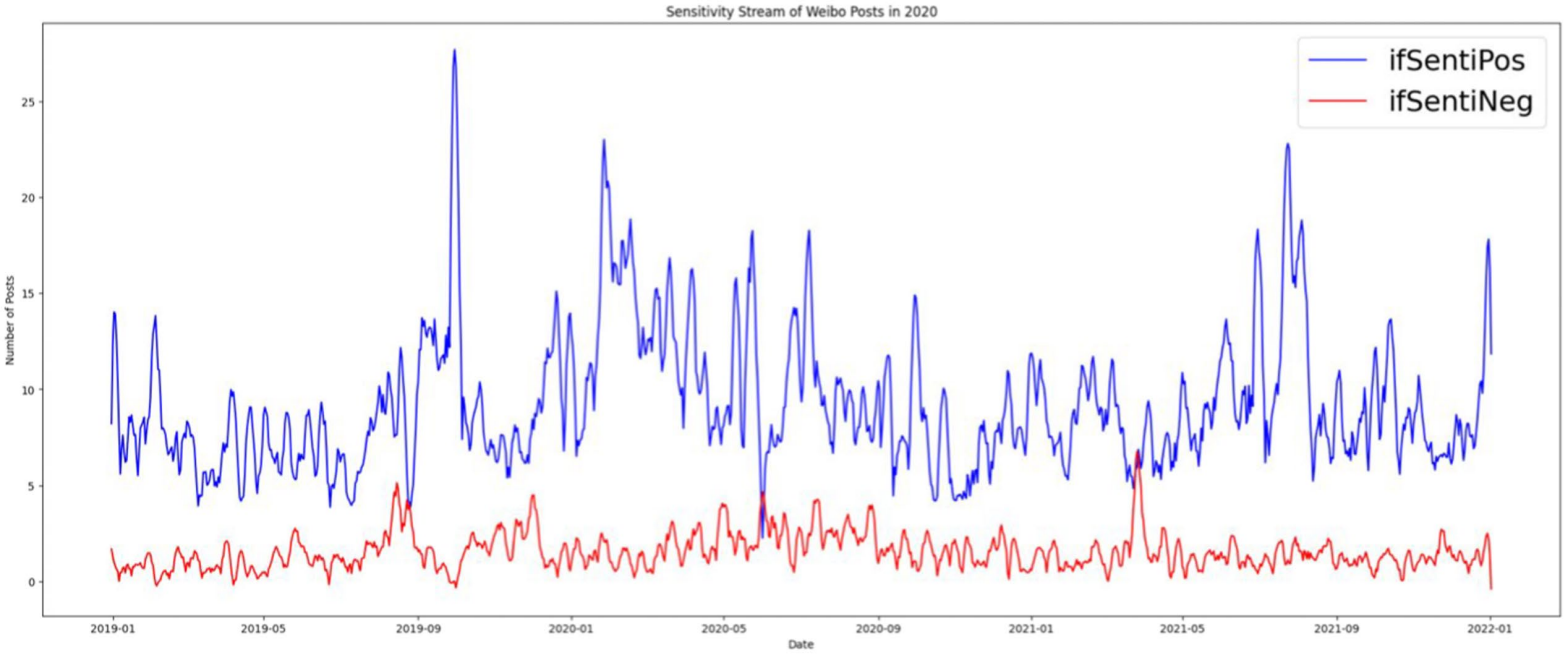
Positive and negative sentiment on *People’s Daily* Weibo account from 2019 to 2021.

Traditionally, emotions are irrelevant to hard news about serious topics such as international trade and domestic and foreign politics (Tuchman, 1973). By contrast, soft news is expected to be emotional, sensational, and personality centered (Patterson, 2000; Plasser, 2005). We expected a positive association between soft news and emotionality in news posts. Given that soft news was the dominant news format on the *People’s Daily* Weibo account, we expected that news with emotions would also dominate. However, we observed that nonemotional news was dominant except for in October 2019 during the 70th anniversary of the founding of the People’s Republic of China. The state-run media reduced the importance of emotion to cultivate an air of objectivity. However, emotional mobilization occurred throughout, and we observed that most posts contained positive emotion. The number of posts with negative sentiments only exceeded the number with positive sentiments in September 2019, June 2020, and April 2021, which corresponds to a high point for the Hong Kong protests, the rise of criticism and speculation about the origins of SARS-CoV-2 in China, and disputes over Xinjiang cotton, respectively. Those are occasions in which *People’s Daily* either published articles attacking China’s critics or defending Chinese governmental policies against external criticism, and negative sentiment was used to reinforce the critical rhetoric in the posts (Figures 7, 8).

**Figure 9:**
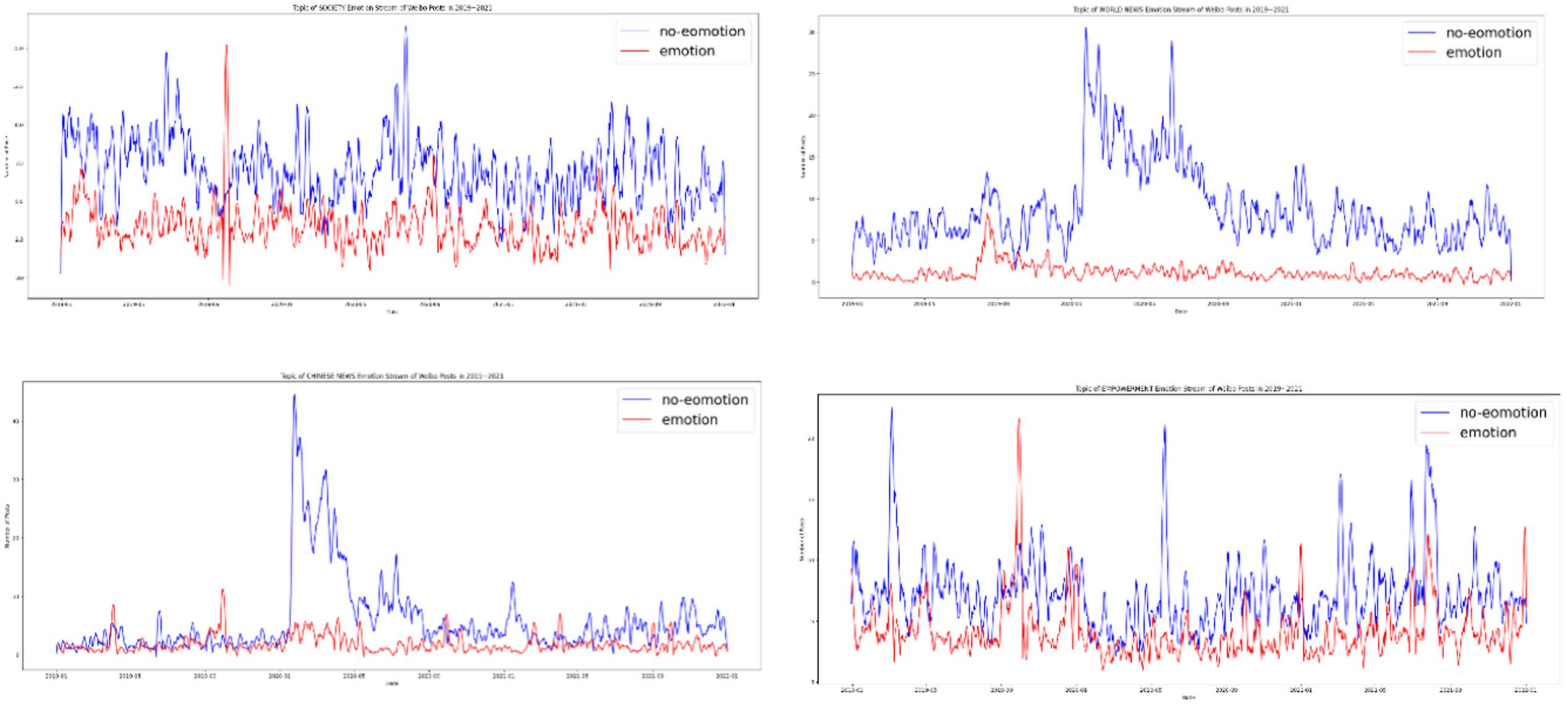
Emotionality of news over time by topic; upper left, society; upper right, world news; lower left, Chinese political news; lower right, positive energy posts.

**Figure 10:**
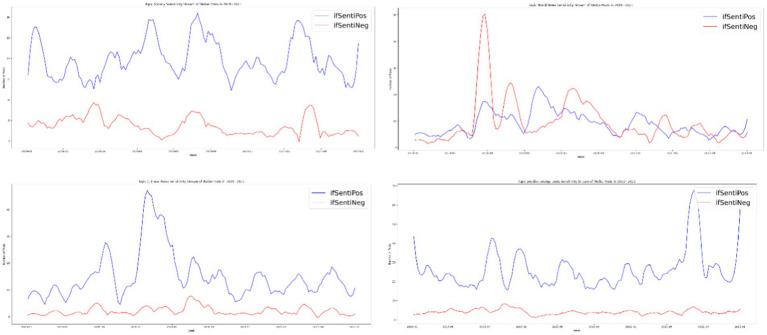
Positive and negative sentiment in the news by topic; upper left, society; upper right, world news; lower left, Chinese political news; lower right, positive energy posts.

Positive-energy posts designed to stimulate optimism and resilience contained the highest density of emotions. Less emotional forms of news were social news, Chinese political news, and world news, in descending order. We examined not only the relationship between emotions and the topics of posts but also the relationship between the valence of emotions and post topics. Social news, Chinese political news, and positive energy posts were more likely to be linked to positive sentiments. *People’s Daily* tended to present Chinese political news with a positive sentiment. By contrast, news related to foreign countries tended to include both positive and negative sentiments. Although both positive and negative news about foreign countries increased during the early stages of the pandemic, positive news appeared at a higher frequency. This positivity in world news was due to coverage of foreign countries’ expressions of prayers, good wishes, and foreign aid for China. However, starting in March 2020, the tone of world news turned negative until July. The main reason is that during this period, critiques of China’s early-stage mismanagement of the pandemic appeared along with investigative reports on the source of COVID-19. The *People’s Daily* Weibo shifted from being a positive energy machine to defending the Chinese government, including defending governmental policies from both domestic and international criticism. At the same time, positive domestic news increased as *People’s Daily* attempted to counter negative external criticism. When the stabilized, the amount of positive news returned to prepandemic levels. In addition, we observed that the number of positive-energy posts decreased dramatically. Overall, we observed that *People’s Daily* preferred to post positive news about China, whereas the tone of international news depended on the event (Figures 9, 10).

We observed three indicators of the softening of the Chinese party press’s digital strategy. First, we observed that soft news that focused on nonpolitical or long-term topics or framed political news from a dramatic, sensational, and human interest–based perspective dominated the *People’s Daily* Weibo account. This indicates that the Chinese state media’s digital strategy softened as the market logic of appealing to the audience prevailed over the party logic of conveying authoritative news information. Second, we detected a predominance of positive-energy posts. Posts that stimulated positive emotions such as comfort, excitement, gratitude, and warmth were widespread on the *People’s Daily* Weibo posts. Positive-energy posts were highly likely to be presented in the form of soft news, including that regarding Chinese domestic social news. Even for news about foreign countries and Chinese domestic politics, almost half of the posts were presented as soft news. The third marker of the softening of the Chinese state media’s digital strategy is the embeddedness of emotions. By contrast with the notion that Chinese digital news is often considerably emotional, we observed that the majority of posts were emotionally neutral. This means that although China’s state media integrated sentiment into its social media content, posts without emotions were still dominant. Although qualitative studies have tended to identify the mobilization of both positive and negative emotions in China’s news posts, our research demonstrated that positive emotions were dominant among sentimental posts. Chinese domestic stories were given positive coverage, whereas foreign countries received mixed treatment, with the tone varying by perspective on China.

## Discussion

Drawing on a longitudinal study of the *People’s Daily* Weibo account, this study solved three puzzles. First, we mapped the Chinese state media’s digital strategies from 2019 to 2022. The time frame enabled us not only to perform an empirical analysis of trends but also to determine the effects of COVID-19 on China’s digital propaganda strategies. Second, our research responded to the increase in scholarship on China’s soft propaganda in the digital era. Unlike case studies that have revealed the dimensions of the transformation of China’s digital propaganda strategy, we used computer-aided textual analysis to validate our results and determine the extent to which China’s digital state media softened. Third, we considered how COVID-19 shaped China’s digital media strategies.

The softening of China’s digital propaganda is an extension of Chinese propaganda’s marketization and commercialization. Our research supported the findings of other studies indicating that Chinese digital propaganda involves social media logic and utilizes soft news and positive energy posts to engage with audiences (Liang, 2019; Zeng and Li, 2021). Thus, the Chinese government used soft propaganda, namely by packaging propagandistic content in nonpolitical entertaining formats (Zou, 2021; Mattingly and Yao, 2022), especially during nonpandemic times. However, our results are inconsistent with the notion that Chinese propaganda is becoming more emotional. Although Zou (2021) suggested that emotional mobilization is increasing in official social media posts, this study finds that the majority of posts are emotional neutral. Posts only contained strong emotions to refute criticism when China faced external or domestic discursive attacks. Emotional mobilization is more likely to help stabilize the Chinese government’s control when that control is under threat (Zhang and Jamali, 2022). Therefore, emotional mobilization is a tool for the Chinese government to distract the public when the Chinese government is challenged. As expected, domestic political news received positive coverage, but foreign news received mixed coverage. When foreign countries expressed friendly diplomatic signals, Chinese digital official media tended to give them positive coverage. When foreign countries adopted antagonistic attitudes toward China, they gave them negative coverage. Therefore, the role of the Chinese state-run media on any platform remains maintaining social stability, defending national interests, and strengthening the government’s position (Li, 2019, p. 145; Wang, 2012).

Studies have suggested that the Chinese state-run media mobilized emotion during COVID-19 (Zou, 2021; Zhang, 2022). The results partially support this notion. We observed that emotional mobilization was used but not in the majority of posts during the pandemic. COVID-19 caused the Chinese official media to return to releasing authoritative information, temporarily reduce the amount of soft news, and release hard news and nonemotional news. This may be because the Chinese government relied on official media to inform the public of the severity of the pandemic and the necessary response measures (Han et al., 2021). This indicates that *People’s Daily* was sought to balance being the legacy media of a party press and a popular social media account. However, the uptick in hard news did not last long. When the pandemic came under control, soft news and positive-energy posts regained dominance. Overall, Chinese propaganda combines hard and soft propaganda to maintain its authority, market relevance, and credibility.

## Conclusion

The internet has changed the digital practices and propaganda model of the Chinese state-run media, especially during the COVID-19 pandemic (Zhang, 2022). This study determined that COVID-19 strongly affected the Chinese state-run media’s practices on Weibo; however, the effects were short lived. Chinese state-run media benefit considerably from soft propaganda, and the changes during COVID-19 were only an emergency measure taken by the Chinese government. Chinese state media have developed a diverse toolbox and applied nuanced measures ranging from emotional mobilization and distraction to timely updates to manage domestic public opinion. Further research may benefit from qualitative content analysis to elucidate how news posts on different topics are packaged as soft or hard news and how emotions are embedded in various posts, especially positive-energy posts. Although this study suggest that emotional mobilization is not significant, however, its overall tendency is increasing. Hence, it could be interesting to explore how the state-led media mobilize emotion on social media through a qualitative analysis. This study could also be extended by combining content analysis with interview (staffs at state-led media). A major question that remains is the extent to which the softening of Chinese digital propaganda was influenced by organizational culture of Chinese state-led media.

## Data availability statement

The original contributions presented in the study are included in the article/Supplementary material; further inquiries can be directed to the corresponding authors.

## Author contributions

All authors listed have made a substantial, direct, and intellectual contribution to the work and approved it for publication.

## Funding

The authors also would like to acknowledge their gratitude for funding from Ministry of Science and Technology “Knowledge Graph of China Studies: Knowledge Extraction, Graph Database, Knowledge Generation” (MOST 110-2628-H-003-002-MY4, Ministry of Science and Technology). The research also receives financial support from China Postdoctoral Science Foundation (Grant number: 2022M712941).

## Conflict of interest

The authors declare that the research was conducted in the absence of any commercial or financial relationships that could be construed as a potential conflict of interest.

## Publisher’s note

All claims expressed in this article are solely those of the authors and do not necessarily represent those of their affiliated organizations, or those of the publisher, the editors and the reviewers. Any product that may be evaluated in this article, or claim that may be made by its manufacturer, is not guaranteed or endorsed by the publisher.

## Footnotes

^1^These materials are available upon request from the corresponding author.
