# Supplementary material for: The softening of Chinese digital propaganda: Evidence from the People’s Daily Weibo account during the pandemic
Source: Front Psychol. 2023 Feb 28;14:1049671. doi: 10.3389/fpsyg.2023.1049671 (PMC10013709; doi:10.3389/fpsyg.2023.1049671)
Supplement: Supplementary file 1 [file Data_Sheet_1.docx]

Appendix A.

|  | Dictionary |
| --- | --- |
| Positive | 加油，爱，相信，好消息，暖，致敬，心疼，辛苦，感恩，感谢，谢谢，牵挂，祝福，平安，感动，开心，光荣，热爱， 爱情，微笑，拥抱，鼓励，戳泪，告慰，安心，白衣天使，白衣战士，可爱，同胞，阻击战，逆行者，快乐，友善，和善，亲密，信赖，宠爱，信任，接纳，感动，开心，信心 (Come on, love, believe, good news, warm, salute, distressed, hard, grateful, thankful, thankful, concerned, blessed, peaceful, moved, happy, glorious, loving, love, smile, hug, encourage, poke tears, console, Peace of mind, white-clothed angel, white-clothed warrior, lovely, compatriot, sniper, retrograde, happy, friendly, kind, intimate, trusting, pampering, trusting, accepting, moving, happy, confident) |
| Negative | 造谣，污蔑，坚决反对，荒谬，威胁，谴责，政治病毒，傲慢，虚伪，甩锅，谣言，抹黑，祸心，信口雌黄，双重标准，霸权，出卖，无能，港独，愤怒 烦躁，嫉妒，生气，不平，愤恨，不满，委屈，沮丧，失望，轻视，轻蔑，讨厌，憎恶，担忧，发怒，焦急，惧怕，哀 、怒 、惧 ，担忧、猜疑、嫉妒，恐惧 ，愤怒，敌意，怒骂，怒，鄙视，愤怒，惨 (Rumors, slander, resolute opposition, absurd, threats, condemnation, political virus, arrogance, hypocrisy, blame, rumors, smears, mischief, slander, double standards, hegemony, betrayal, incompetence, Hong Kong independence, anger, jealousy, anger, Injustice, resentment, dissatisfaction, grievance, frustration, disappointment, contempt, contempt, hatred, hatred, worry, anger, anxiety, fear, sorrow, anger, fear, worry, suspicion, jealousy, fear, anger, hostility, scolding, anger, contempt, anger, miserable) |
